# The IHCA paradox: high early mortality, yet comparable long-term survival in STEMI patients

**DOI:** 10.1007/s11739-026-04372-4

**Published:** 2026-07-24

**Authors:** Aneta Aleksova, Alessandra Lucia Fluca, Milijana Janjusevic, Giulia Vanin, Gioachino Di Carlo, Giulia Barbati, Filippo Maria Rubbo, Leonardo Viotto, Kenneth Pesenti, Maria Marketou, Daniela Aschieri, Alberto Peratoner, Carlo Pegani, Andrea Perkan, Gianfranco Sinagra, Giuseppe Ristagno

**Affiliations:** 1 Department of Medical, Surgical and Health Sciences, University of Trieste, Via Valdoni 7, 34129 Trieste, Italy; 2 Cardiothoracovascular Department, Azienda Sanitaria Universitaria Giuliano Isontina, Trieste, Italy; 3 Department of Medical Surgical and Health Sciences, Biostatistics Unit, University of Trieste, Via Valdoni 7, 34129 Trieste, Italy; 4 Cardiology Department Crete, School of Medicine, Heraklion University General Hospital, University of Crete, Crete, Greece; 5 Cardiologic Unit, Ospedale Guglielmo da Saliceto Piacenza, Piacenza, Italy; 6 Emergency Medical Service, Azienda Sanitaria Universitaria Giuliano Isontina, Trieste, Italy; 7 Department of Pathophysiology and Transplantation, University of Milan, Milan, Italy; 8 Fondazione IRCCS Ca’ Granda Ospedale Maggiore Policlinico, Milan, Italy

**Keywords:** STEMI, IHCA, ROSC, In-hospital survival, Prognosis

## Abstract

In-hospital cardiac arrest in STEMI (STEMI-IHCA) patients poses a formidable mortality risk, yet comprehensive survival data remain scarce. This study compared clinical characteristics and outcomes of STEMI-IHCA versus non-cardiac arrest (non-CA) patients. A cohort of 3311 STEMI patients, treated in the Cath lab and transferred to the intensive cardiac care unit, was retrospectively analyzed over a 21-year period (2003–2024). Of these, 208 (6.3%) had experienced STEMI-IHCA. The mean age was 66.2 ± 12.6 years; 77.8% were male. STEMI-IHCA patients more frequently had peripheral artery disease, multivessel disease, and renal impairment. In contrast, non-CA patients had higher rates of dyslipidemia and smoking. A striking 72.7% of STEMI-IHCA cases presented with a shockable rhythm, achieving rapid ROSC (< 1 min).Despite this, in-hospital mortality for STEMI-IHCA was drastically higher (25.5 vs. 2.8% for non-CA, *p* < 0.01), with most deaths occurring within three days (66 vs. 44.2%, *p* < 0.01). Predictors of in-hospital mortality were IHCA, older age, peripheral artery disease, reduced LVEF, and higher TIMI risk index. Among patients discharged alive, long-term mortality over a 96-month follow-up was similar between STEMI-IHCA and non-CA patients (36.8 vs. 33.3%, *p* = 0.37), confirmed by Kaplan–Meier analysis (*p* = 0.73) as well. Our findings confirm the high risk of in-hospital mortality risk associated with STEMI-IHCA. Nevertheless, among patients discharged alive, particularly with rapid ROSC, the long-term prognosis can align remarkably with that of STEMI patients who did not experience IHCA. This underscores the critical importance of prompt intervention as a powerful determinant of favorable long-term outcomes.

**Graphical abstract:**

**Figure:**
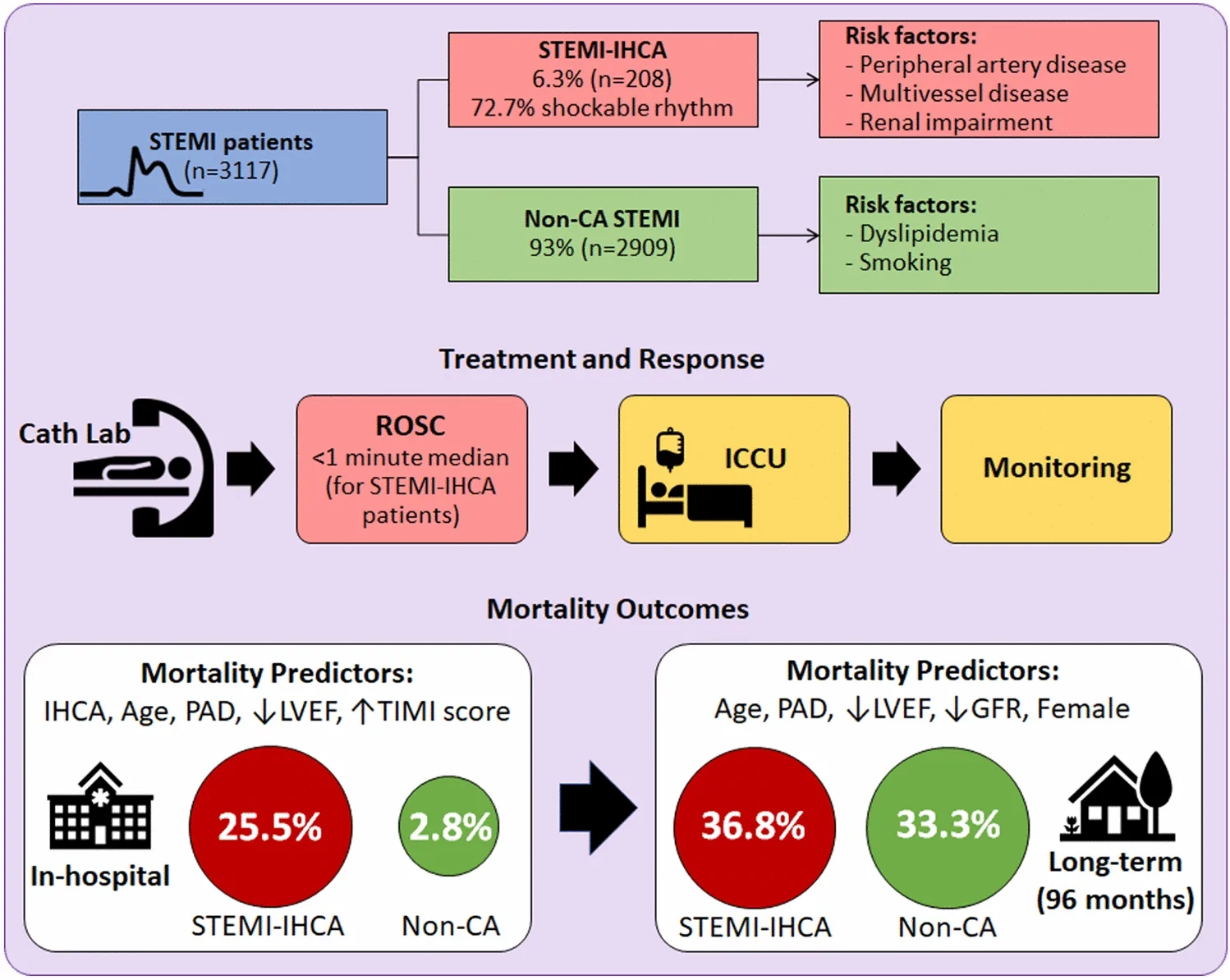

## Background

ST-elevation myocardial infarction (STEMI) represents a critical and life-threatening manifestation of coronary artery disease (CAD), demanding immediate reperfusion therapy to minimize myocardial damage and improve patient survival [1, 2]. Despite significant advancements in acute coronary syndrome management, including rapid diagnosis, pharmacological interventions, and percutaneous coronary intervention (PCI), STEMI patients remain at a substantial risk for severe major adverse events [1].

Among these critical complications, in-hospital cardiac arrest (IHCA) is particularly devastating, adding another layer of complexity and significantly worsening the prognosis [3, 4]. This specific subgroup of patients presents unique challenges in terms of diagnosis, management, and subsequent care [3, 4]. Unfortunately, comprehensive data on the incidence, clinical profiles, and long-term outcomes of IHCA occurring in patients with STEMI (STEMI-IHCA) remain limited [3, 4].

Indeed, the true burden of IHCA in the STEMI population is difficult to ascertain due to significant data limitations [5]. While general IHCA incidence is reported in large national and international registries, these often fail to capture the nuanced context of cardiac arrest specifically within the STEMI population [5]. Furthermore, variations in institutional reporting practices, definitions of IHCA events, and the specific coding of complications within STEMI cohorts hinder the aggregation of robust, comparable datasets [6]. General data on IHCA, such as the reported incidence of 6–7 per 1000 hospital admissions in the United States, or the 1.5–2.8 per 1000 admissions reported in Europe, do not accurately reflect the specific risk profile and outcomes associated with STEMI-related cardiac arrest [7, 8]. It is crucial to note that while these general IHCA data are informative, survival and risk profiles can vary markedly depending on the underlying cause of cardiac arrest [5].

This study, therefore, aims to address critical gaps in the management of patients with STEMI complicated by IHCA. We examined the incidence of IHCA, delineated distinct clinical profiles, including demographic characteristics, comorbidities, and acute presentation features that contribute to its occurrence, and provided a comprehensive analysis of in-hospital outcomes, long-term survival, and quality of life following this severe event. Through this detailed exploration, we seek to provide valuable insights to enhance the identification, prevention, and management of IHCA in this vulnerable population.

## Methods

### Study population, definitions, end point, and follow-up

A single-center retrospective study was conducted on consecutive patients with STEMI treated in the cardiac catheterization laboratory at the University Hospital of Trieste, Italy, between December 1st, 2003, and December 31st, 2024. STEMI-IHCA was defined according to Utstein guidelines [9] and could occur at any time during hospitalization, in any hospital setting, including the catheterization laboratory or the intensive cardiac care unit (ICCU). Patients with out-of-hospital cardiac arrest were excluded. Clinical data were collected throughout the entire hospital stay and in-hospital mortality was recorded regardless of when it occurred. Clinical data were retrieved from the Cardionet and G2 electronic health record systems (INSIEL, Trieste, Italy). Variables collected encompassed medical history, clinical presentation, time to return of spontaneous circulation (ROSC), as well as outcomes including in-hospital survival and mortality both during hospitalization and follow-up. Given that IHCA events occurred in patients undergoing continuous 24‑h monitoring, we defined T_0_ as the first recorded moment of cardiac arrest, and the endpoint as the first evidences of ROSC with interruption of resuscitation. Time to ROSC was rounded down to the nearest minute for data‑entry purposes; thus, a time to ROSC value of zero corresponded to a duration ranging from 0 to 59 s.

All-cause mortality was designated as the primary endpoint. Follow-up duration was determined from the date of hospital discharge until either death or the study endpoint on April 8th, 2025. Full follow-up data were obtained for every patient, with no losses reported. This study was approved by the local ethics committee (N°67/2015, updated CEUR‐2021‐Em‐220 on 22/06/2021, PROT 0025024/P/GEN/ARCS), adheres to the Declaration of Helsinki and is currently ongoing.

### Statistical analysis

Statistical analyses were carried out using IBM^®^ SPSS Statistics (version 24) and R package (version 4.2.2), employing the “survival” and “rms” libraries. The distribution of continuous variables was assessed using the Kolmogorov–Smirnov test. Data are summarized as mean ± standard deviation (SD) for normally distributed variables or as median with interquartile range (IQR) otherwise. Group comparisons for continuous variables were performed using one-way analysis of variance (ANOVA) for parametric data and the Mann–Whitney *U* test for non-parametric data. Categorical data were expressed as percentages and either the Chi-square test or Fisher’s exact test were used for group comparison, as appropriate.

We created a categorical variable to evaluate the incidence of IHCA and mortality, stratifying the study period into four-time intervals: 2003–2008, 2009–2014, 2015–2019, and 2020–2024. Comparisons between categorical groups were performed using the Chi-square test.

To explore factors associated with in-hospital mortality, univariable binary logistic regression was initially performed. Variables demonstrating a *p* value < 0.05 in the univariable analysis were incorporated into a multivariable logistic regression model using a backward stepwise approach. Selection of covariates was further supported by clinical judgment and existing literature.

Survival analyses, landmarked at hospital discharge and restricted to patients discharged alive, were conducted using the Kaplan–Meier method, with group comparisons assessed by the log-rank test. Univariable Cox proportional hazards regression was used to estimate the effect of individual predictors, followed by construction of a multivariable Cox regression model using backward stepwise selection for variables meeting the *p* value < 0.05 in the univariable stage.

Hazard ratios were reported with 95% confidence intervals (CI). A *p* value < 0.05 was considered statistically significant for all analyses.

## Results

### Baseline characteristics

Among the 3311 patients with STEMI included in the cohort, the mean age was 66.2 ± 12.6 years, and 77.8% were male. The most prevalent cardiovascular risk factors were hypertension (60.3%), dyslipidemia (58.8%), and smoking (47.6%). Baseline characteristics of the study population are summarized in Table 1.

**Table 1:** Baseline characteristics of the study population stratified by the occurrence of in-hospital cardiac arrest (IHCA)

	Overall (*n* = 3311)	STEMI (*n* = 3103)	STEMI-IHCA (*n* = 208)	*p* value
Age (years)	66.2 ± 12.6	66.2 ± 12.5	67.4 ± 13.4	0.18
Male gender (%)	73.6	73.6	73.6	0.99
Previous AMI (%)	12.4	12.5	12.5	0.99
Hypertension (%)	60.3	60.3	59.1	0.73
Family history (%)	25	25.4	19.7	0.07
Diabetes mellitus (%)	20.8	20.8	21.2	0.9
Smoking (%)	47.6	48.1	40.4	0.03
Dyslipidemia (%)	58.8	59.3	51.4	0.03
Peripheral artery disease (%)	5.7	5.2	13.5	< 0.01
SBP (mmHg)	132.4 ± 28.9	133.9 ± 28.2	115.6 ± 30.8	< 0.01
DBP (mmHg)	75 [65–84]	76 [66–85]	70 [60–80]	< 0.01
Heart rate (bpm)	74.9 ± 18.1	74.1 ± 17.7	83.9 ± 21.1	< 0.01
Killip > I (%)	23.6	22.1	45.2	< 0.01
TIMI risk index (point)	24 [17–32]	23 [17–31]	30 [21–49]	< 0.01
eGFR (ml/min/1.73 m^2^)	75.4 [60–91.2]	76.1 [60.9–92]	65.9 [52.6–84.2]	< 0.01
History of eGFR < 60 ml/min/1.73 m^2^ (%)	24.9	23.8	38.1	< 0.01
Anemia (%)	33.2	33	34.8	0.63
Multivessel disease (%)	44.1	43.5	53.1	0.03
LVEF (point %)	50.9 ± 10	51.2 ± 10	45.8 ± 9.2	< 0.01
Aspirin (%)	95.6	96.5	85.8	< 0.01
Clopidogrel (%)	60.2	61.3	48.6	< 0.01
Prasugrel (%)	24.2	24.9	16.8	0.01
Ticagrelor (%)	21.6	23.2	15.1	0.02
Beta-blockers (%)	78.4	79.6	65.6	< 0.01
ACE-I/ARB (%)	72.5	74.1	55.4	< 0.01
Nitrates (%)	25.8	26.3	21	0.11
Statins (%)	87.8	89.4	71.4	< 0.01

*ACE-I* angiotensin converting enzyme inhibitors, *AMI* acute myocardial infarction, *ARB* angiotensin receptor blockers, *DBP* diastolic blood pressure, *eGFR* estimated glomerular filtration rate, *IHCA* in-hospital cardiac arrest, *LVEF* left-ventricular ejection fraction, *SBP* systolic blood pressure, *STEMI* ST-elevation myocardial infarction, *TIMI *thrombolysis in myocardial infarction

A total of 208 patients (6.3%) experienced IHCA, of whom 56 (27.3%) had an unshockable rhythm. The median time to ROSC was 0 [0–1] min. In our cohort, STEMI-IHCA patients more frequently had peripheral artery disease, multivessel disease, and renal impairment, and tended to present with lower admission blood pressure and higher heart rate (all *p* < 0.01); whereas baseline age and prevalence of major cardiovascular risk factors were similar.

**Fig. 1:**
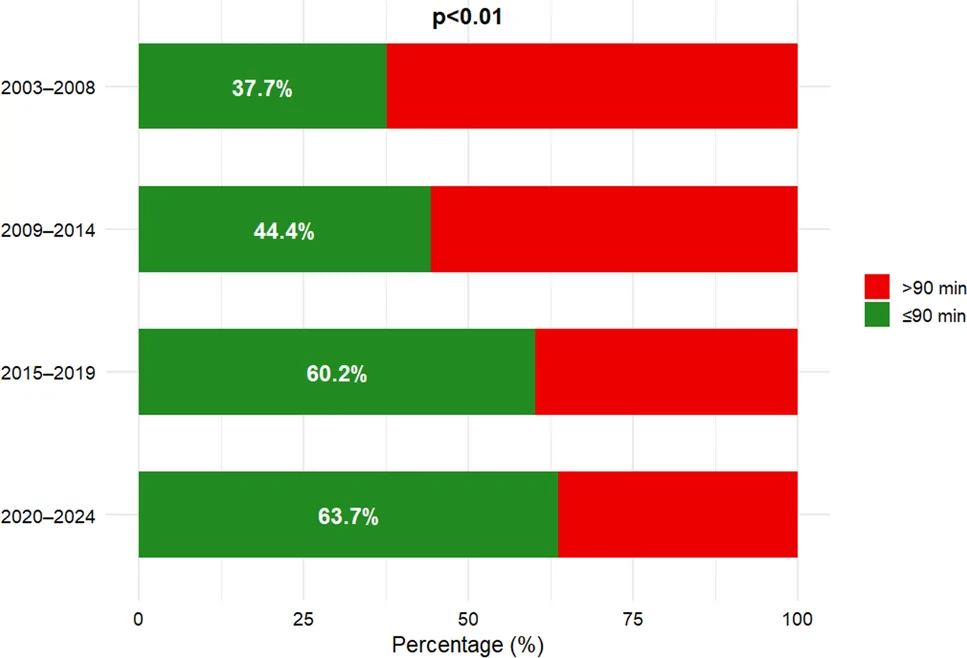
Distribution of primary PCI timing across four temporal groups. Bars represent the percentage of patients treated within 90 min (green) and after 90 min (red)

The incidence of STEMI-IHCA was 10.6% in 2003–2008 and significantly decreased over time (*p* < 0.01), reaching 4.3% in 2009–2014, 6.7% in 2015–2019, and 4.7% in the most recent period 2020–2024. In 2003–2008, only 37.7% of patients had PCI ≤ 90 min, rising to 44.4% in 2009–2014, 60.2% in 2015–2019, and 63.7% in 2020–2024 (*p* < 0.01) (Fig. 1).

### In-hospital outcome

Overall, 139 patients (4.2%) died during hospitalization, after a median hospital stay of 3 [0–10] days. A significant decrease in in-hospital mortality was observed through the years (*p* < 0.01). Compared to survivors, in-hospital non-survivors were older and more frequently had significant comorbidities such as diabetes mellitus (DM) and peripheral artery disease (all *p* < 0.01). They also presented with a worse initial clinical presentation, including higher Killip class, a lower blood pressure, a higher heart rate, and a significantly reduced left-ventricular ejection fraction (LVEF) (all *p* < 0.01) (Table 2). Significantly higher in-hospital mortality for STEMI-IHCA patients was observed compared to uncomplicated STEMI (25.5 vs. 2.8%, respectively, *p* < 0.01). Moreover, early mortality within the first three days was also significantly higher in the STEMI-IHCA group (66 vs. 44.2%, *p* = 0.01). Cardiovascular complications were the leading cause of early death in most STEMI-IHCA patients.

**Table 2:** Baseline characteristics of patients with STEMI stratified by in-hospital outcome

	Discharged alive (*n* = 3172)	In-hospital death (*n* = 139)	*p* value
Age (years)	65.8 ± 12.5	77.4 ± 9.5	< 0.01
Male gender (%)	74.3	56.1	< 0.01
Previous AMI (%)	12.3	15.2	0.32
Hypertension (%)	60.1	63.8	0.39
Family history (%)	25.8	8.7	< 0.01
Diabetes mellitus (%)	20.3	32.6	< 0.01
Smoking (%)	48.5	27.5	< 0.01
Dyslipidemia (%)	59.5	43.9	< 0.01
Peripheral artery disease (%)	5.1	20.3	< 0.01
SBP (mmHg)	133.6 ± 28.2	108.6 ± 32.6	< 0.01
DBP (mmHg)	76 [66–85]	60 [50–73]	< 0.01
Heart rate (bpm)	74.3 ± 17.5	86.5 ± 25.6	< 0.01
Killip > I (%)	21	82	< 0.01
TIMI risk index (point)	23 [17–31]	49 [34–67]	< 0.01
eGFR (ml/min/1.73 m^2^)	76.3 [61.3–92]	49.9 [35.3–65.8]	< 0.01
History of eGFR < 60 ml/min/1.73 m^2^ (%)	22.9	68.9	< 0.01
Anemia (%)	32.5	46.2	0.01
Multivessel disease (%)	43.3	62.6	< 0.01
LVEF (point %)	51.2 ± 9.8	38 ± 14.7	< 0.01
Aspirin (%)	96.3	71.3	< 0.01
Clopidogrel (%)	60.8	40.7	< 0.01
Prasugrel (%)	25.1	1.2	< 0.01
Ticagrelor (%)	22.4	7.8	0.01
Beta-blockers (%)	80.3	14.5	< 0.01
ACE-I/ARB (%)	74.1	17.7	< 0.01
Nitrates (%)	26.1	19.4	0.23
Statins (%)	89.5	29.5	< 0.01

Tables report baseline and discharge variables

Intercurrent events such as IHCA are handled in regression models

*ACE-I* angiotensin converting enzyme inhibitors, *AMI* acute myocardial infarction, *ARB* angiotensin receptor blockers, *DBP* diastolic blood pressure, *eGFR* estimated glomerular filtration rate, *IHCA* in-hospital cardiac arrest, *LVEF* left-ventricular ejection fraction, *SBP* systolic blood pressure, *STEMI* ST-elevation myocardial infarction, *TIMI* thrombolysis in myocardial infarction

On logistic regression analysis, experiencing an IHCA remained an independent predictor of in-hospital mortality along with older age, peripheral artery disease, worse LVEF and higher TIMI risk index, renal function, after adjustment for gender, multivessel disease, primary PCI within 90 min, DM, and renal function (Table 3).

**Table 3:** Logistic regression analysis for predictors of in-hospital death

	OR [95% CI]	*p* value
Age (per 5 year increase)	1.53 [1.25–1.88]	< 0.01
Peripheral artery disease (yes vs no)	1.97 [1.27–3.07]	< 0.01
IHCA (yes vs no)	5.72 [2.61–12.53]	< 0.01
LVEF (per 5 points % decrease)	1.58 [1.35–1.83]	< 0.01
TIMI risk index (per 2 points increase)	1.05 [1.01–1.09]	0.01

The table reports only significant predictors

The model was adjusted for gender, multivessel disease, primary PCI within 90 min, eGFR < 60 ml/min/1.73 m^2^, diabetes mellitus, and estimated glomerular filtration rate

*IHCA* in-hospital cardiac arrest, *LVEF* left-ventricular ejection fraction, *TIMI* thrombolysis in myocardial infarction

### Long-term outcome

**Fig. 2:**
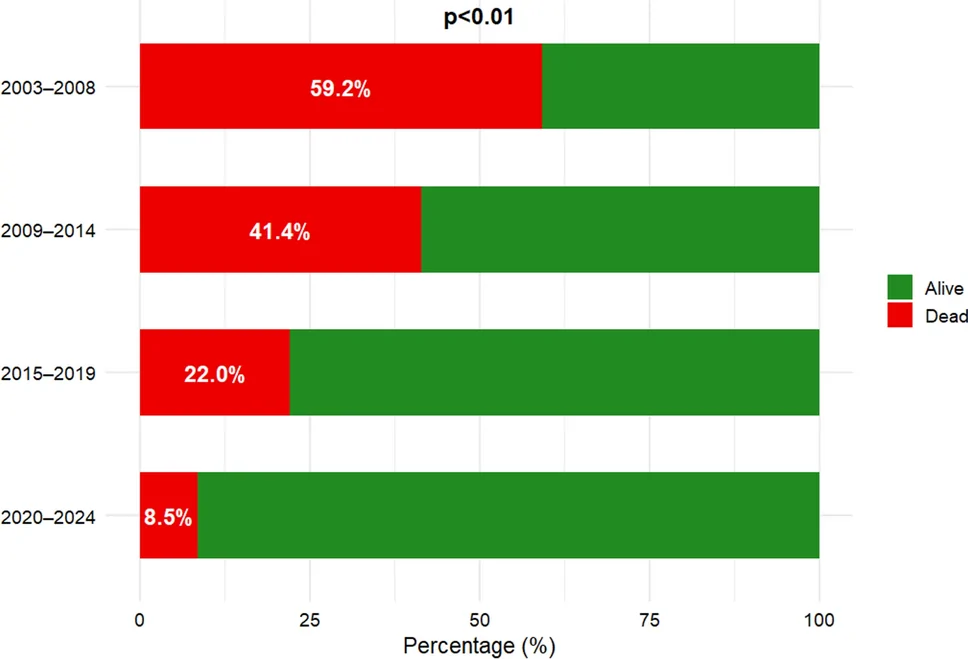
Distribution of mortality at follow-up across four temporal groups. Bars represent the percentage of patients alive (green) and dead (red) at follow-up within each group

Compared to survivors, patients who died during follow-up were older, less frequently male, and more likely to have major comorbidities, such as DM, hypertension, peripheral artery disease, anemia, renal impairment, and multivessel disease (all *p* < 0.01). They also presented with a worse clinical profile, including higher heart rate, lower diastolic pressure, and markedly reduced LVEF (all *p* < 0.01). Over the years, a steady decrease in mortality was observed (*p* < 0.01) (Fig. 2).

**Fig. 3:**
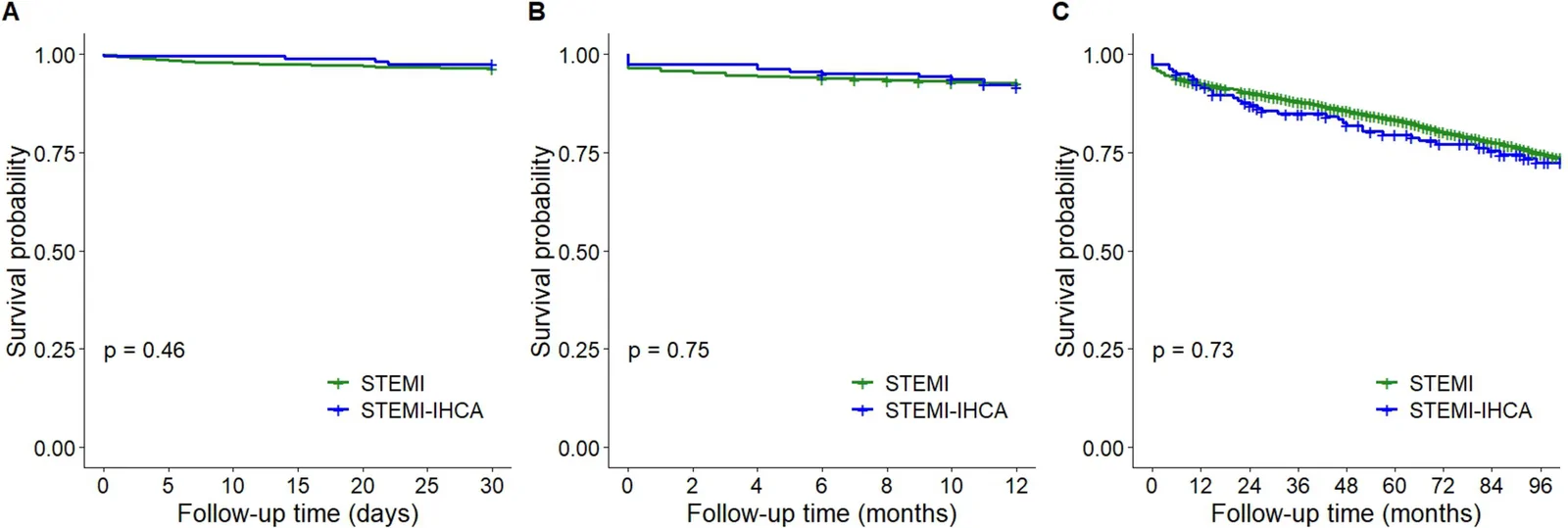
Kaplan–Meier survival curves comparing patients with STEMI and STEMI-IHCA. **A **30-day survival, **B **1-year survival, and **C **long-term survival over the entire follow-up period

Kaplan–Meier analysis showed no significant difference in survival between patients with STEMI-IHCA and those with uncomplicated STEMI at 30 days (*p* = 0.46) and after 1 year (*p* = 0.75) (Fig. 3a, b).

Over the entire follow-up period of 96 ± 65 months, 1090 (33.5%) patients died. Patients who died during follow-up were older and more often had hypertension, DM, peripheral artery disease, and a history of previous myocardial infarction (all *p* < 0.01). They also showed worse renal function and lower LVEF (both *p* < 0.01) (Table 4).

**Table 4:** Baseline characteristics of patients with STEMI stratified by all-cause mortality during follow-up

	Alive (*n* = 2168)	Died (*n* = 1090)	*p* value
Age (years)	62.5 ± 11.9	73.2 ± 10.6	< 0.01
Male gender (%)	77.8	65.3	< 0.01
Previous AMI (%)	10.1	17	< 0.01
Hypertension (%)	54.9	71.1	< 0.01
Family history (%)	29.5	17.4	< 0.01
Diabetes mellitus (%)	16.5	28.7	< 0.01
Smoking (%)	50.9	42.2	< 0.01
Dyslipidemia (%)	59.2	59.1	0.96
Peripheral artery disease (%)	2.1	12	< 0.01
SBP (mmHg)	133.1 ± 27.5	132.8 ± 29.8	0.79
DBP (mmHg)	76 [67–85]	75 [62.3–82]	< 0.01
Heart rate (bpm)	73.3 ± 16.7	76.5 ± 19.5	< 0.01
Killip > I (%)	31.7	100	< 0.01
TIMI risk index (point)	20 [14–25]	30 [23–39]	< 0.01
eGFR (ml/min/1.73 m^2^)	81.6 [68.2–96.2]	65.1 [50.8–79.9]	< 0.01
History of eGFR < 60 ml/min/1.73 m^2^ (%)	13.8	40.9	< 0.01
Anemia (%)	29	37.4	< 0.01
Multivessel disease (%)	40.7	50.2	< 0.01
LVEF (point %)	51.7 ± 9.5	42.6 ± 11.9	< 0.01
Aspirin (%)	96.6	95.7	0.26
Clopidogrel (%)	48	80.5	< 0.01
Prasugrel (%)	35.5	9.1	< 0.01
Ticagrelor (%)	24.4	16.1	0.01
Beta-blockers (%)	83.7	73.9	< 0.01
ACE-I/ARB (%)	73.6	73.7	0.95
Nitrates (%)	17.4	39.2	< 0.01
Statins (%)	94.2	81.7	< 0.01

*ACE-I* angiotensin converting enzyme inhibitors, *AMI* acute myocardial infarction, *ARB* angiotensin receptor blockers, *DBP* diastolic blood pressure, *eGFR* estimated glomerular filtration rate, *IHCA* in-hospital cardiac arrest, *LVEF* left-ventricular ejection fraction, *SBP* systolic blood pressure, *STEMI* ST-elevation myocardial infarction, *TIMI* thrombolysis in myocardial infarction

No significant difference in mortality was observed between STEMI-IHCA and uncomplicated STEMI (36.8% vs. 33.3%, *p* = 0.37). Furthermore, Kaplan–Meier analysis showed no difference in cumulative survival probability between the two groups (*p* = 0.73, Fig. 3c) or based on initial presentation rhythm (*p* = 0.05).

At multivariable Cox regression analysis, experiencing an IHCA was not associated with an increased risk of long-term mortality (*p* = 0.71). In contrast, older age, female gender, peripheral artery disease, lower LVEF, and impaired renal function were independent predictors of all-cause mortality, after adjusting for history of prior ischemic events, hypertension, DM, smoking, peripheral artery disease, multivessel disease, and AMI location (anterior, inferior, or lateral) (Table 5).

**Table 5:** Cox regression analysis for predictors of all-cause mortality

	HR [95% CI]	*p* value
Age (per 5 years increase)	1.48 [1.25–1.77]	< 0.01
Peripheral artery disease (yes vs no)	2.71 [1.35–5.43]	< 0.01
LVEF (per 5 points % decrease)	1.54 [1.37–1.73]	< 0.01
eGFR < 60 ml/min/1.73m^2^ (yes vs no)	2.12 [1.37–3.82]	0.01
Female vs male	1.82 [1.01–3.26]	0.04

The table reports only significant predictors

The model was adjusted for IHCA (*p* = 0.71), history of prior ischemic events, hypertension, diabetes mellitus, smoking, multivessel disease, and acute myocardial infarction location

*eGFR* estimated glomerular filtration rate, *IHCA* in-hospital cardiac arrest, *LVEF* left-ventricular ejection fraction

## Discussion

The findings of the current study shed critical light on the long-term prognosis of patients with STEMI-IHCA. While it is well established that IHCA in the setting of STEMI carries a formidable in-hospital mortality burden, with our study’s findings aligning with this high immediate risk, our unique contribution lies in demonstrating that survivors of IHCA during STEMI can achieve long-term survival outcomes comparable to STEMI patients who did not experience cardiac arrest. This observation is a significant departure from the prevailing understanding, as much of the existing literature often underscores the persistently high mortality associated with IHCA during the follow-up. Our data, spanning a substantial 21-year period and showing an exceptionally rapid median time to ROSC less than 1 min among STEMI-IHCA patients, strongly suggests that prompt and highly effective acute in-hospital resuscitation efforts can fundamentally alter the long-term trajectory for this critically ill population.

Cardiac arrest, encompassing both out-of-hospital and in-hospital events, is reported to complicate approximately 5–10% of STEMI presentations [10].

However, when focusing specifically on STEMI-IHCA patients, reported incidences from large administrative datasets tend to be lower. For instance, a large nationwide study in the United States, analyzing nearly 4 million STEMI admissions between 2000 and 2007, reported an IHCA incidence of 2.6% [10]. Similarly, data from a Chinese registry including 40,670 STEMI patients found an IHCA incidence of 2.2% [3]. By contrast, our study reported an IHCA incidence of 6.3% among STEMI patients, which is notably higher from these large administrative datasets. Examining the temporal evolution of STEMI-IHCA within our cohort, a significant reduction was observed, with the proportion decreasing from approximately 10% in the early 2000s to around 5% in the most recent period. This declining trend likely reflects advancements in STEMI treatment and overall patient management over the years. However, despite this internal reduction, the incidence in our cohort remains higher compared to other registries. This difference is likely attributable to several factors.

First, the continuous evolution and optimization of pre-hospital emergency medical services and faster transport times mean that patients who, in earlier eras or in less-optimized regional systems, might have suffered fatal cardiac arrest before reaching the hospital, are now more frequently arriving alive but critically unstable. This increased “salvage” of severe out-of-hospital events effectively shifts the location of cardiac arrest from pre-hospital to in-hospital, thereby increasing the observed IHCA incidence, particularly in a high-acuity tertiary center like ours that serves as a referral hub for such complex cases. Second, the progressive implementation of rapid reperfusion strategies likely contributed to the observed decline in STEMI-IHCA incidence over time [3]. Third, although our study is retrospective, the use of detailed clinical records from a single tertiary center ICCU may have enabled more accurate and granular identification of IHCA. Large nationwide administrative databases often rely on broader coding practices, which can lead to underreporting or misclassification of transient or rapidly resolved IHCA events, contributing to lower reported incidences [6, 11]. Fourth, since our study included only STEMI patients admitted to the ICCU, these represent a higher-risk population with more severe disease, multivessel involvement, and a greater burden of comorbidities, thus increasing the risk of cardiac arrest.

Analyzing the cohort according to the presence of cardiac arrest, it was noted that STEMI-IHCA patients, despite similar baseline age and prevalence of cardiovascular risk factors compared to non-CA STEMI patients, had more coronary artery disease, more comorbidities, and worse initial clinical presentation. These findings suggest that STEMI-IHCA patients represent a uniquely vulnerable subgroup characterized by a more extensive and advanced systemic atherosclerotic burden, coupled with acute hemodynamic instability indicative of severe myocardial insult [3, 12]. The lack of differentiation by traditional risk factors underscores the presence of other critical underlying vulnerabilities, such as a greater degree of physiological fragility, chronic inflammation, and oxidative stress, which predispose these individuals to catastrophic acute events. This highlights the urgent need for comprehensive risk stratification tools that integrate markers of systemic vascular health and physiological resilience to enable earlier and more precise identification for targeted preventive strategies.

The devastating immediate impact of IHCA on STEMI patients is consistently reported across various cohorts, often exceeding 50%. Indeed, large cohort studies have reported in-hospital mortality rates of 49–53% for STEMI-IHCA patients [3, 10]. This high mortality persists despite the fact that IHCA events in monitored settings, such as the ICCU, typically have shorter response times and are more likely to be witnessed, allowing for rapid intervention [13].

However, survival after IHCA is not determined solely by response time. Other critical factors include the presenting rhythm, duration of resuscitation, and underlying patient comorbidities. Certainly, shorter time to ROSC is strongly associated with improved survival and better neurological outcomes [2, 14]. We speculated that exactly for the remarkably short median time to ROSC observed in our cohort, the in-hospital mortality for STEMI-IHCA patients was 26.3%. This near-immediate restoration of spontaneous circulation is paramount in mitigating the systemic damage, particularly neurological injury, that typically follows cardiac arrest [15]. Indeed, the American College of Cardiology, American Heart Association, and collaborating societies highlight that favorable prognostic features, such as witnessed arrest, shockable rhythm, and rapid ROSC, are key determinants of survival in patients with STEMI patients and cardiac arrest [2, 14]. Observational data and risk models consistently show that prolonged time to ROSC (> 30 min) is associated with markedly reduced survival [2, 16]. Moreover, survival is also higher when cardiac arrest is due to an acute myocardial infarction or ischemia, compared to non-cardiac causes such as advanced heart failure [5].

Nevertheless, despite this comparatively favorable immediate outcome, STEMI-IHCA remains a profoundly severe complication: we still observed a nearly tenfold higher in-hospital mortality among STEMI-IHCA patients (26.3%) compared to uncomplicated STEMI patients (2.7%), and a markedly high early mortality within the first three days (67.3%), both of which are in line with the severe prognosis typically reported for this condition. Our analysis of the overall STEMI cohort revealed that patients who died in-hospital had a distinct clinical profile compared to survivors, characterized by a higher burden of pre-existing risk factors. Specifically, deceased patients were notably older [17–20] and presented with a higher prevalence of significant comorbidities, including DM and peripheral artery disease [21, 22]. Crucially, they also exhibited markers of poorer hemodynamic and cardiac status upon admission, which likely reflects the severity of the initial myocardial injury. These included higher Killip class, lower blood pressure, higher heart rate, and a significantly reduced LVEF. This is particularly relevant to the STEMI-IHCA group, where the devastating impact of cardiac arrest is likely compounded by a higher prevalence of these same adverse characteristics [3, 23]. Indeed, while the logistic regression analysis confirmed that experiencing an IHCA is a powerful independent predictor of mortality, it also identified these underlying conditions as equally critical determinants of a fatal outcome. Therefore, these characteristics serve as vital clinical indicators for identifying patients at exceptionally high risk.

Finally, our follow-up data suggest that once patients survive the index hospitalization, their long-term prognosis becomes similar to that of patients without IHCA. These findings are consistent with multiple large cohort studies demonstrating that while IHCA in STEMI is associated with markedly increased in-hospital and 30-day mortality, among those who survive to hospital discharge, long-term mortality (beyond 30 days) is not significantly different from STEMI patients without cardiac arrest [24, 25]. However, it should be noted that the observed similarity in long-term outcomes may partly reflect survivor bias, as patients with more reversible pathology are more likely to survive the acute phase and a return to baseline physiological stability. Besides this, favorable long-term outcomes among IHCA survivors are particularly likely in patients with rapid ROSC, preserved neurologic function (e.g., regained consciousness after resuscitation), shockable initial rhythms, and timely successful revascularization [24–28]. Prolonged periods of cerebral ischemia are the primary drivers of poor neurological outcomes and contribute significantly to overall mortality and morbidity in cardiac arrest survivors [15]. The ability to intervene within seconds at the bedside, or in monitored settings ICCU or cardiac catheterization labs where resources and trained personnel are readily available, likely plays a crucial role in improving long-term prognosis.

Our data not only confirm these findings with precision but also provide novel insights through a comprehensive 21-year long-term follow-up. These are encouraging findings for both clinicians and patients, as they support the necessity of an appropriate in-hospital management and structured post-arrest rehabilitation strategies. In light of this, from 2015 onwards, international guidelines have increasingly emphasized the importance of structured post-resuscitation care, recognizing that survival after a cardiac arrest does not depend solely on the acute event, but also on comprehensive in-hospital management and post-arrest rehabilitation strategies [8, 29]. These guidelines recommend a multidisciplinary approach, including neurological and cardiological evaluation to prevent complications [8].

This study has several limitations that should be acknowledged. First, its retrospective and single-center design may limit the generalizability of our findings to other institutions or populations with different organizational models of care. Second, the study period spans 21 years during which significant changes occurred in guidelines and post-arrest care protocols, which might have introduced heterogeneity in the cohort. Third, information on the cause of death was not available. Despite these limitations, the study also has several strengths. In particular, the inclusion of all consecutive STEMI patients admitted to the ICCU over a 21-year period minimizes selection bias and reflects a real-world population in a tertiary center. Furthermore, our follow-up extends beyond previously published data and we have also provided information about time to ROSC in the IHCA setting.

## Conclusions

In conclusion, our study clearly confirms the markedly high risk of in-hospital mortality associated with STEMI-IHCA. However, among patients discharged alive, especially when ROSC is rapidly achieved, as in our cohort, the long-term prognosis was similar to STEMI patients not experiencing cardiac arrest. These findings highlight the pivotal role of timely and effective in-hospital management, not only in reducing immediate mortality but also in securing a long-term survival comparable to that of uncomplicated STEMI, thereby supporting the need for structured post-arrest care and rehabilitation strategies. Therefore, there is the urgent need for timely recognition and proactive management of patients at risk to mitigate these poor short-term outcomes.

## Data Availability

All relevant data are included within the article.

## Abbreviations

ACC: Acute coronary care
ANOVA: Analysis of variance
CAD: Coronary artery disease
DM: Diabetes mellitus
ICCU: Intensive cardiac care unit
IHCA: In-hospital cardiac arrest
IQR: Interquartile range
LVEF: Left-ventricular ejection fraction
PCI: Percutaneous coronary intervention
ROSC: Return of spontaneous circulation
SD: Standard deviation
STEMI: ST-elevation myocardial infarction
STEMI-IHCA: ST-elevation myocardial infarction complicated by in-hospital cardiac arrest
